# Sustained Suppression of Gorlin Syndrome-Associated Basal Cell Carcinomas with Vismodegib or Sonidegib: A Case Series

**DOI:** 10.3390/curroncol30100661

**Published:** 2023-10-16

**Authors:** Raquel Wescott, Wolfram Samlowski

**Affiliations:** 1 School of Medicine, University of Nevada, Reno, NV 89557, USA; rwescott@med.unr.edu; 2 Comprehensive Cancer Centers of Nevada (Medical Oncology), Las Vegas, NV 89148, USA; 3 Department of Medicine, Kirk Kirkorian School of Medicine, University of Nevada Las Vegas (UNLV), Las Vegas, NV 89106, USA

**Keywords:** nevoid basal cell carcinoma syndrome, sonidegib, vismodegib, hedgehog inhibitors, Gorlin syndrome, PTCH mutation

## Abstract

Nevoid basal-cell carcinoma syndrome (Gorlin syndrome) is characterized by numerous cutaneous basal cell carcinomas mediated by mutations in the hedgehog pathway. Vismodegib or sonidegib represent promising treatment options. We identified 10 Gorlin patients who were treated with sonidegib (*n* = 6) or vismodegib (*n* = 4) between March 2012 and March 2022. We analyzed the activity, toxicity, and duration of the response to oral hedgehog inhibitors. The number of new tumors that developed prior to treatment or after treatment as well as the time of response and durability of responses were assessed. All patients achieved a complete remission. With a 30.7 ± 48.4-month median follow-up, the drug treatment significantly reduced the number of new basal cell cancers from a mean of 28.3 ± 24.6 prior to treatment to a mean of 1.4 ± 2.0 during treatment (*p* = 0.0048). The median time to develop a new basal cell cancer was 47.3 months. Three patients eventually developed localized recurrences. After resection, ongoing treatment suppressed the development of additional lesions. One patient developed numerous new drug-resistant basal cell cancers and died of acute leukemia. Six patients required treatment modifications for toxicity. Sustained hedgehog inhibitor treatment can suppress the progression of both new and existing basal cell carcinomas for an extended period. Drug administration schedule adjustments improved tolerance without altering efficacy, potentially contributing to a prolonged response duration.

## 1. Introduction

Gorlin syndrome (nevoid basal-cell carcinoma syndrome) is a rare autosomal dominant genetic condition that causes affected individuals to develop dozens to hundreds of basal cell carcinomas (BCCs) over their lifetime [1]. Few studies on Gorlin syndrome prevalence exist (reviewed by Evans et al. [1]). The most quoted prevalence figure, 1:57,000, comes from a study of a UK population of four million people in northwest England. The potential range is between 1:30,827 and 1:164,000. There is a possible predilection for Caucasians (although this may be due to ascertainment bias related to BCC development, which is less frequent in other races). As expected, there is a similar rate of Gorlin syndrome in both sexes due to autosomal dominant inheritance. Approximately 70–80% of individuals with Gorlin syndrome have a pertinent family history, while about 20–30% result from a de novo pathogenetic variant. The genetic penetrance of *PTCH1* mutations appears to be quite high.

Gorlin syndrome is characterized clinically by the development of numerous BCCs (ranging from nodular to ulcerative plaque morphology) [2], palmar pits, jaw cysts, and skeletal abnormalities [2]. Other associated features may include rib and spine abnormalities and the early calcification of the falx cerebri. Characteristic facial features can include frontal bossing, hypertelorism, macrocephaly, and occasionally a cleft lip and/or palate and other skeletal abnormalities. Less common findings include desmoplastic medulloblastoma during childhood, as well as numerous other neoplasms including ovarian and cardiac fibromas, mesenteric keratocysts, rhabdomyosarcomas, and meningiomas.

A number of criteria have been proposed for the diagnosis of Gorlin syndrome (nevoid basal cell carcinoma syndrome). Note that the diagnostic criteria do not require molecular testing. The diagnosis of NBCCS is established in a proband with the following findings: two major diagnostic criteria and one minor diagnostic criterion or one major criterion and three minor diagnostic criteria [1]. A similar series of diagnostic criteria was proposed by Kimonis et al. [3]. No study has been able to assess which combination of diagnostic criteria represents the best trade-off between sensitivity and specificity.

**Major criteria:**

- Lamellar (sheet-like) calcification of the falx or clear evidence of calcification in an individual younger than 20 years of age. Falx calcification is nearly always present and is visible on anteroposterior (AP) X-rays of the skull after 20 years of age.
- Jaw keratocyst. Odontogenic keratocyst histologically; seen on an orthopantogram as an area of translucency.
- Palmar/plantar pits (≥2); particularly useful in diagnosis and more pronounced when the hands and feet are soaked in warm water for up to ten minutes. Pits may appear as white “punched-out” or pink “pin-prick” lesions.
- Multiple basal cell carcinomas (BCCs) (>5 in a lifetime) or a BCC before 30 years of age. A provision needs to be made for a decreased risk of BCC in individuals with dark skin and an increased risk in those with light skin living in hot, sunny climates, particularly those with type 1 Celtic skin and red hair, and of this group, particularly those with the common MC1R variant (rs1805007), which can modify the age of onset for Gorlin syndrome.
- A first-degree relative with Gorlin syndrome.
- Lympho-mesenteric or pleural cysts.

**Minor criteria:**

- Childhood medulloblastoma (also called primitive neuroectodermal tumor).
- Macrocephaly (OFC > 97th centile).
- Cleft lip/palate.
- Vertebral/rib anomalies observed on chest X-ray and/or spinal X-ray (see Notes regarding radiographs); bifid/splayed/extra ribs; bifid vertebrae.
- Preaxial or postaxial polydactyly.
- Ovarian/cardiac fibromas.
- Ocular anomalies (e.g., cataract, developmental defects, and pigmentary changes in the retinal epithelium).

**Suggestive molecular features:**

- Identification of a heterozygous germline PTCH1 or SUFU pathogenic (or likely pathogenic) variant on molecular genetic testing (see Table 1). This finding establishes the diagnosis if the clinical features are inconclusive.
- Occasional variants in PTCH2 have been found in individuals with NBCCS, but these may not be conclusive [4].

The overwhelming number of lesions in Gorlin syndrome complicates treatment, as not all BCCs can be effectively excised, and patients may require dozens or hundreds of surgical procedures in their lifetime, which may result in extensive scars and disfigurement [5]. Other treatment options are needed.

Recent studies have identified pathogenic mutations in the hedgehog pathway, especially in “patched” (*PTCH1* and *PTCH2*) genes, in Gorlin syndrome [6]. Inhibitors of downstream signaling targeting “smoothened” (*SMO*) have been identified (vismodegib or sonidegib). These agents are commonly referred to as “hedgehog inhibitors” (HHIs). In sporadic basal cell carcinomas, these oral agents have shown significant clinical activity for the treatment of locally advanced or metastatic disease [7,8]. This is because sporadic BCCs usually express non-inherited (somatic) *PTCH* mutations. Vismodegib and sonidegib can cause significant side effects, which include hair loss, taste changes, asthenia, and muscle cramps [9]. While the use of oral HHIs has been studied among patients with sporadic BCCs, there are limited publications concerning the long-term effectiveness and tolerability in patients with Gorlin syndrome. In this case series, we evaluated the effectiveness of oral HHIs in a series of patients with clinically defined Gorlin syndrome.

## 2. Materials and Methods

### 2.1. Subjects and Design

Patients were identified for potential inclusion in this retrospective data analysis via a search of a HIPAA (Health Information Portability and Accessibility Act)-compliant IKnowMed clinical oncology database (McKesson, Houston, TX, USA). Potential records for review were identified by searching for patients treated by one physician (WS) with an oral hedgehog inhibitor (vismodegib or sonidegib) and a diagnosis of multiple basal cell carcinomas between March 2012 and March 2022. Patient records were evaluated for characteristic findings of Gorlin syndrome. The diagnosis of Gorlin syndrome was based on published clinical criteria [1], also including individuals with a strong family history or identified to have germline molecular mutations in the “Hedgehog” pathway. Patients taking vismodegib or sonidegib for other indications, such as sporadic basal cell carcinomas, were excluded from analysis.

### 2.2. Study Methods

Data were extracted onto a spreadsheet (Excel, Microsoft, Redmond, WA, USA), which included demographics (age and date of birth), comorbid conditions, the type of HHI prescribed, and duration of HHI treatment. The initial response to HHI treatment was described, as were the side effects and patterns of drug response/resistance. The number of lesions prior to HHI treatment and the number of lesions during HHI treatment were estimated from pathology reports and dermatologist’s notes. The time to progression was calculated from the start of HHI treatment to the date of documented recurrence. If the patient had no recurrence, the patient was censored at the end of study date (31 May 2022). Additionally, if genetic testing was conducted, the patient’s genotype associated with Gorlin syndrome was recorded. An arbitrary unique patient number was assigned to each patient, and patient data were de-identified prior to analysis. This study design was reviewed by the Western IRB chair and deemed exempt from full IRB review.

### 2.3. Treatment Regimens

Patients with Gorlin syndrome were customarily treated via repeated surgical excisions of the basal cell carcinomas by dermatologists. If the number of lesions was too large for reasonable surgical management, patients were referred to a medical oncologist (WS) for treatment with an oral hedgehog inhibitor. Treatment was initiated with oral agents (vismodegib 150 mg/d or sonidegib 200 mg/d orally). Since these medications are only produced in one fixed-dose tablet size, the schedule of administration was adjusted if intolerable side effects occurred. The initial schedule alteration was a reduction to 5 days/week, then to 3 or even 2 days/week if necessary. A description of toxicity was extracted from each patient’s medical record.

A few patients interrupted treatment with HHIs due to intolerable side effects such as muscle cramps and hair loss, change in insurance, or the co-payment cost of the medication. Lesions sometimes progressed during the period without HHI treatment, and this was not counted when evaluating treatment response. In some patients, radiation was added as part of planned treatment of bulky, neglected lesions, as previously described [10]. One patient was included in a previously published phase II trial, who received 200 mg sonidegib daily for 4 years [11]. This patient continued treatment for an additional 4 years after the conclusion of this clinical trial and is therefore included in the current analysis.

### 2.4. Response Assessment

Response to treatment with HHI was assessed by evaluating the number of lesions while taking the HHI, in comparison to the number of lesions prior to treatment. Complete response was defined as complete resolution of all skin lesions. Partial response was defined as reductions in the size and number of existing lesions, with no new lesions developing. Progression was defined as regrowth of lesions or development of new basal cell skin cancers. Time to progression (TTP) was assessed by the development of biopsy-proven new lesions while taking an HHI. Relapses were defined as local or generalized, determined by the magnitude and location(s) of new or recurrent basal cell carcinomas. Localized resistance was determined to be the development of 1–3 new superficial, resectable lesions while being treated with the HHI, whereas generalized resistance was determined to be the development of dozens or hundreds of multifocal new lesions. Progression-free survival was assessed as the duration of time between the initiation of HHI treatment and the first BCC recurrence.

### 2.5. Statistical Analysis

Descriptive statistics were calculated from an Excel spreadsheet (Microsoft, Redmond, WA, USA). Time to first new lesion was calculated using the method of Kaplan and Meier. Comparison of the number of lesions prior to HHI therapy compared to after HHI treatment was performed via paired Student’s *t*-test.

## 3. Results

We identified 10 patients who met the criteria to be included in this study. The median age was 60.0 ± 13.9 years (±standard deviation). There were seven men and three women. Of these 10 patients, 6 patients were treated with sonidegib, and 4 patients were treated with vismodegib (Table 1). Unfortunately, familial genetic testing was frequently not covered by insurance; therefore, only four of the patients were screened for mutations in *PTCH1*. Testing for mutations in other potentially relevant HHI pathway genes, including *PTCH2*, *SMO*, *GLI1*, and *SUFU*, was not routinely performed. Two of the patients were identified to have *PTCH1* mutations, while the other two genetically tested patients did not have mutations in *PTCH1*. Two additional patients had a family history with a documented *PTCH1* mutation but could not themselves undergo confirmatory testing.

**Figure 1:**
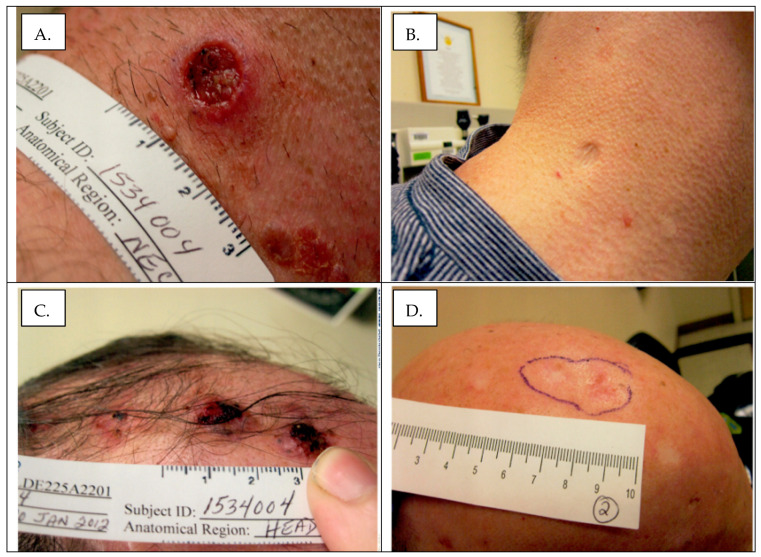
Treatment response in patient 2 (of >20 lesions). Panel (**A**): Neck lesion pretreatment. Panel (**B**) Neck lesion after 2 years of therapy. Note scar remodeling. Panel (**C**): Scalp lesions prior to treatment. Panel (**D**): Scalp lesions after 2 years of therapy. Note development of global alopecia.

In all patients, the initial response to the treatment with an HHI was the complete disappearance of all BCCs within 3 months from the start of the HHI treatment. A typical durable response is shown (shown in Figure 1). Four patients eventually developed a delayed recurrence: three of these developed a small number (1–2) of localized new tumors that were controlled via surgical excision (Table 2). One patient had a multifocal progression with dozens of new BCCs and a concomitant development of acute myelogenous leukemia. The patient died due to rapid leukemia progression. This patient’s progressing BCC lesions did not respond to the HHI retreatment.

Treatment-related side effects were common. The most common side effects were muscle cramps (*n* = 7), followed by hair loss/thinning (*n* = 6), taste changes (*n* = 5), weight loss (*n* = 3), nausea (*n* = 3), vomiting (*n* = 1), insomnia (*n* = 1), stomach upset (*n* = 1), amenorrhea (*n* = 1), and asthenia (*n* = 1). One patient developed long-term hair loss on sonidegib, which has been previously reported as an adverse event of vismodegib [12]. One patient temporarily discontinued HHI treatment due to muscle cramps. Two patients temporarily discontinued treatment due to copayment costs or a change in insurance, allowing for new lesions to develop. Schedule modifications were required for six patients due to side effects, resulting in amelioration of toxicity and allowing for the continuation of treatment (Table 2).

**Figure 2:**
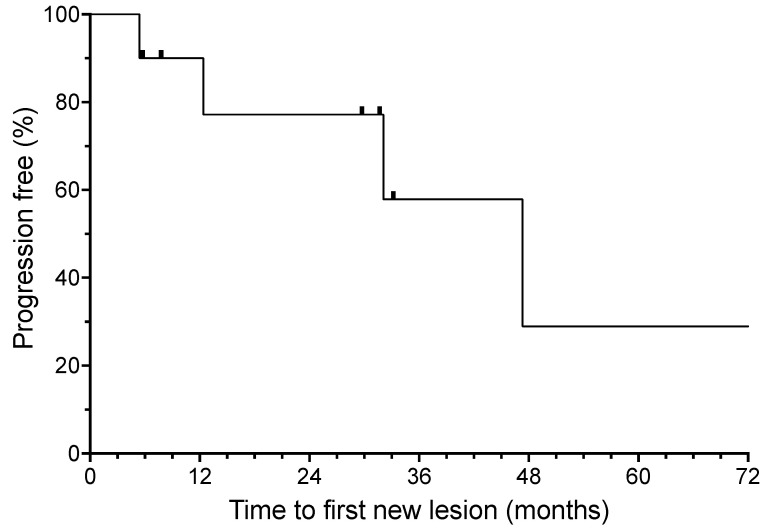
Time to first new basal cell carcinoma from the initiation of oral hedgehog inhibitor (HHI) therapy. Hash marks indicate censored patients.

The median time for the development of a new lesion (time to progression) was 47.3 months (shown in Figure 2). For the three patients with a localized recurrence of BCC during treatment, the median time to develop a second new lesion after the resection of the isolated recurrence was over 12.4 months at the time of analysis. All three of these patients currently continue to benefit from ongoing HHI therapy.

Patients who developed a BCC recurrence during HHI therapy generally had a prior history of a larger number of resected lesions prior to the initiation of HHI treatment. They also tended to have been diagnosed at a younger age. The median age at diagnosis for those who developed a recurrence was 10.5 years, and the median age at diagnosis for those who did not have a recurrence was 48 years. Of the two patients with documented *PTCH1* mutations, both experienced a lengthy complete response from the onset of HHI therapy. Three patients required superficial electron beam radiation therapy in addition to concurrent HHI treatment to ablate large pre-existing lesions [10].

**Figure 3:**
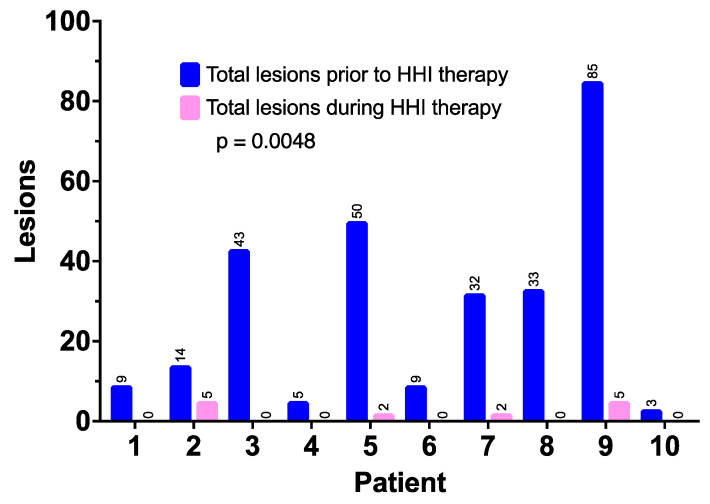
New basal cell carcinomas prior to the start of HHI therapy and during ongoing treatment.

HHI treatment also resulted in a significant reduction in new BCCs in all patients when compared to the pretreatment (shown in Figure 3). The number of BCCs per patient prior to the HHI treatment was a mean of 28.3 (range of 3–85 documented BCCs), whereas the mean number of new BCCs during treatment (with median potential follow-up of over 30 months) was 1.4 (range of 0–5) (*p* = 0.0048).

## 4. Discussion

In humans, there are three “hedgehog” (*HH*) peptide family members (Sonic, Desert, and Indian hedgehog) that play important roles in growth and development in different organ systems (reviewed by Sari et al. [13]). The apparent differences in the functions of these soluble proteins are due to the diverse tissue regulated patterns of expression. The *HH* pathway also appears to play an essential role in the maintenance of somatic stem cells and pluripotent cells [13].

All three *HH* isoforms function by binding to “patched” (*PTCH*), a 12-pass transmembrane receptor. Two mammalian *PTCH* homologs have been identified: Patched1 (*PTCH1*) and Patched2 (*PTCH2*). Both isoforms bind all three *HH* peptides with equal affinity, and each inhibits the activity of the *SMO* protein but differs in tissue expression, thus modulating diverse cellular development patterns. Mutations of the *PTCH* gene have been demonstrated in several diseases such as basal cell nevus syndrome (BCNS), nevoid basal cell carcinoma syndrome, and sporadic basal cell carcinomas. Medulloblastomas tend to be associated with “suppressor of fused homologue” (*SUFU)* gene mutations [1]. The interaction of unbound *PTCH* isoforms with *SMO* destabilizes *SMO*, resulting in increased lysosomal degradation. In the absence of *SMO* activation, *SUFU* binds *GLI* family proteins in the cytoplasm, preventing their activation as transcription factors [13].

*SMO* activation (release from *PTCH* inhibition via ligand binding or inactivating *PTCH* mutations) results in the release of *GLI* 1, 2, and 3 proteins, allowing for their entry into the nucleus, where *GLI* acts as a transcription. factor. There is developing evidence that *GLI1* activation increases cellular apoptosis and DNA damage [6]. Thus, mutations in the *HH* signaling pathway can lead to genetic instability and cancer development (reviewed in Onodera et al. [6]).

The tumorigenesis in BCC is ligand-independent as the pathway is constitutively activated via mutations in the components of the *HH* pathway, including activating mutations in the *SMO* or inactivating mutations in the *PTCH1* or *SUFU* [1]. In Gorlin syndrome, a *PTCH1* mutation on chromosome 9 is frequently identified. The monoallelic inactivation of *PTCH* is enough to trigger oncogenic activity. It should be noted that 27% of clinically defined Gorlin patients do not have a detectable mutation in *PTCH1* or *SUFU*, suggesting that other unidentified mutations are responsible [1].

About 90% of sporadic BCC patients have an identifiable loss-of-function mutation in at least one allele of *PTCH1*, 30% have biallelic inactivation, and the remaining 10% have activating gain-of-function mutations in *SMO*. In sporadic, but not Gorlin syndrome-related BCCs, the biallelic inactivation of *PTCH1* is identified in about 30% of patients. Increased *PTCH1* and *GLI* protein expressions can be detected in most human BCCs [14]. It is therefore believed that the upregulation of *HH* signaling is a key driver in all BCCs, leading to the uncontrolled proliferation of basal cells in the skin.

Cyclopamine, an *HH* signaling inhibitor, was the first drug that was used to treat BCCs. This drug binds to *SMO*, inhibiting this receptor and downstream signaling (reviewed in Cucci et al. [15]). Unfortunately, cyclopamine causes serious fetal malformations. Since then, safer synthetic cyclopamine derivatives have been developed as *HH* pathway inhibitors. These agents also have better pharmacological and inhibitory properties. The approved agents include vismodegib (GDC-0449) and sonidegib (LDE225). Sekulic reported the long-term outcome of oral vismodegib (150 mg/d) treatment for locally advanced BCCs (laBCCs), also including rare metastatic BCC patients (mBCC) [7]. The objective response via an investigator assessment in this trial was 60.3% in the locally advanced BCC group (20 patients had a complete response and 18 patients had a partial response). In this trial, the investigator-assessed response rate was 48.5% in the mBCC group. In the mBCC group, all patients had partial responses. The median progression-free survival was 26.2 months in the laBCC group and 14.8 months in the mBCC group. The median overall survival was not reached in the laBCC cohort and was 33.4 months in the mBCC cohort. Thirty-three deaths (31.7%) were reported in the trial; all were judged to be related to age, tumor progression, or comorbid illnesses. None of the deaths were related to vismodegib toxicity. Patients who received treatment for ≥12 months had higher rates of muscle spasms, alopecia, dysgeusia, weight loss, fatigue, and nausea as side effects. This led to treatment discontinuation in over 25% of the patients.

Dummer reported the long-term outcome of a trial comparing 200 and 800 mg/day oral dosing of sonidegib in the laBCC and mBCC groups [11]. In this trial, the objective response rate (by central review) was 56% for the laBCC group and only 8% for the mBCC patients in the 200-mg/d group. In the 800 mg/d group, the objective response was 46.1% for the laBCC patients and 17% for the mBCC patients. The median PFS was 26.09 months for the la BCC group and 23.96 months for the mBCC group. In the 800 mg/d group, the median DOR was 23.3 months. The most common reasons for sonidegib discontinuation were AEs, seen in 23 (29%) and 57 (377%) patients. AEs in the 200-mg group were primarily grade 1 or 2. Grade 3–4 AEs related to treatment were reported in 25 (32%) and 65 (43.3%) patients receiving 200 mg and 800 mg of sonidegib, respectively. Most AEs were manageable and reversible with dose interruptions or reductions. One (1%) and seven (47%) deaths occurred within 30 days of the study treatment for the 200 mg and 800 mg groups, respectively; none of the deaths were suspected to be related to the study drug. Overall, the most common AE by the preferred term was muscle spasms, which were reported in 43 (54%) and 104 (693%) patients in the 200 mg and 800 mg groups, respectively. Alopecia was grade ≤ 2, reported in 39 (49%) and 87 (580%) patients receiving 200 mg and 800 mg of sonidegib, respectively. Thus, the 200 mg/d dose received regulatory approval.

We identified a number of case reports and three published case series that included Gorlin syndrome patients treated with HHI [16,17,18,19,20,21,22,23,24]. These studies each demonstrated a rapid onset of response and a reduction in the number and size of lesions. The durability of the responses and tolerability of long-term treatment has not been well characterized.

In another real-world experience with sonidegib, Nazzaro et al. described 11 patients, including 4 patients who were diagnosed with Gorlin syndrome [25]. Seven (63.6%) patients experienced adverse events (AEs), but only three patients discontinued therapy due to toxicity. Four patients (50%) achieved biopsy-confirmed complete clinical remission (CR). A partial response (PR) was found in three patients out of eight (37.5%). One patient (12.5%) had stable disease (SD). All four patients suffering from basal cell nevus syndrome achieved disease control by being treated with sonidegib. The long-term follow up and toxicity management are not described.

Our patient series suggests that HHI treatment induces more prolonged complete responses in patients with Gorlin syndrome than what is reported in patients with sporadic BCCs. This is potentially due to “oncogene addiction” related to inherited mutations in the hedgehog pathway [26]. It has been suggested that new lesion development in Gorlin syndrome requires the development of additional resistance mutations in *PTCH1* [20,27]. Unfortunately, this possibility could not be evaluated in our patient series. In the small number of patients that progressed while undergoing HHI treatment, the development of resistance appeared to be a localized phenomenon, allowing for the resection of isolated new lesions with an ongoing benefit from the continuation of HHI therapy. We hypothesize that this may be due to the clonal development of a drug resistance mutation.

Usually, the treatment of unresectable BCCs with HHI is continued until disease progression. HHI toxicity frequently leads to treatment discontinuation [9]. Periodic drug holidays have been suggested to improve tolerability [28]. While several of our patients experienced side effects initially, schedule modifications allowed for the resolution of side effects in most patients and allowed for the long-term continuation of treatment. These schedule modifications did not appear to reduce efficacy and still resulted in a marked suppression of new BCC development. The improved tolerability of HHI therapy may have contributed to a longer median time to a first new lesion (47.3 months) in our patients.

In our series, patients who developed recurrences tended to have been diagnosed with Gorlin at a younger age and had a higher mean number of lesions prior to treatment compared to those who did not develop recurrences. The cause of this increased risk of progression is uncertain and could be due to more extensive early-life sun damage, differing genetic mutation patterns, or other undescribed factors. A better understanding of the phenotype–genotype correlation seems important.

Due to the activities of vismodegib and sonidegib in BCCs, including Gorlin syndrome-associated tumors, a number of new HHI agents have been tested (reviewed in Jain et al. [29] and Villani et al. [30]). These include topical agents such as patigedib and agents directed against drug resistance mutations (taladegib, TAK441, and LEQ506). In addition, PD-1-directed monoclonal antibodies (cemiplimab) have shown significant clinical activity against sporadic basal cell cancers [31]. The role of these newer agents in the treatment of Gorlin patients is still being evaluated.

Preclinical studies have suggested that untreated BCCs demonstrate an “immunosuppressed tumor microenvironment (TME) when *HH* signaling is active (reviewed in Gambini et al. [32]). This can lead to accumulation of T-reg cells and increased expressions of immune checkpoint inhibitory molecules including programmed death (PD)-1/PD-ligand 1. Patient biopsies before and after 4 weeks of treatment with an *HH* inhibitor showed a significative change in the TME after therapy, characterized by the disruption of the immune privilege and the promotion of a local adaptive immune response. HHI therapy appeared to upregulate MHC class I expression and increase infiltrating immune cells (CD8+, CD4+, HLA-DR-class II+ mononuclear cells, and CD68+ macrophages) at the cancer site. An increased infiltration of tumors with T-regs was also observed, along with chemokine gene induction. Thus, improved anti-tumor immunity could potentially play a role in HHI treatment responses.

The limitations in the current study include the small sample size in a rare disorder and a relatively short duration of follow-up for three patients. We were limited in our ability to perform confirmatory genetic testing. There was significant variability between patients in the number of BCCs diagnosed prior to treatment. This could result in a lead-time bias. Additionally, there was potential ascertainment bias in determining the number of lesions prior to and during treatment. New lesions were identified from pathology reports or dermatology records. As is frequent with non-melanoma skin cancer, the lesions may have been excised or obliterated without biopsies being performed or without the pathology being evaluated. In addition, due to the lengthy period in which patients were at risk for BCC development, records were frequently not available for longer than 5–7 years. The patients in our series also frequently changed dermatologists, making it difficult to identify all prior biopsy records.

## 5. Conclusions

This study focused on the outcome of 10 patients with clinically defined Gorlin syndrome who were treated with oral HHIs. We examined the efficacy of HHIs in reducing BCCs, side effects, and increasing progression-free survival among this population. All 10 patients experienced a rapid onset of complete clinical response after only 3 months of treatment. Moreover, among the 10 patients, the number of newly developing BCCs was significantly decreased during HHI treatment compared to pretreatment (*p* = 0.0048), with 6 (60%) patients never experiencing a recurrence during ongoing HHI therapy. The duration of responses was quite lengthy if dosing adjustments were made to minimize toxicity. While four (40%) patients experienced a recurrence, three were able to undergo an additional limited resection(s), resulting in an additional protracted period of disease control with continuing HHI therapy. One patient developed generalized drug resistance and concomitant acute leukemia and did not respond to HHI rechallenge.

Future studies should continue to investigate HHI treatment outcomes among patients with Gorlin syndrome with a longer follow-up. The use of long-term HHI therapy to suppress new BCCs in Gorlin appears very promising. As eventual resistance may be due to resistance mutations [20], the development of additional HHI inhibitors that can overcome this form of resistance may also be helpful. The resection of potentially resistant lesions in patients with small numbers of progressive lesions is recommended, as this may extend the durability of benefit from HHI therapy.

## Figures and Tables

**Table 1:** Patient demographics.

UPN	Age	Sex	Genotype	FH BCCs	Associated Clinical Manifestations	Comorbid Conditions
1	60	M	PTCH1 W387 *	FH BCCs	>20 active BCCs	ADHD
2	46	M	ND ^†^	FH BCCs ^†^	>20 active BCCs Cysts in jaw, palmar pits, shallow hip sockets	None
3	57	F	ND	None	>10 active BCCs Palmar pits, jaw cysts, odontogenic cysts	Hypothyroidism, lower back pain
4	70	M	No *PTCH1* mutation	FH BCCs	>5 active BCCs Lipomas Sister with ovarian CA	AF, lipoma, DVT, antiphospholipid antibody syndrome
Plea5	30	F	ND	FH BCCs, Father had jaw cysts	>2 BCCs Palmar pits, jaw cyst	Chronic pain syndrome, nausea
6	64	M	PTCH 1 splice site 2561-1G>A	None	>5 active BCCs	HTN, anxiety disorder, nausea
7	42	F	ND ^†^	FH BCC ^†^	>5 active BCCs Jaw cysts, ovarian cysts, hydrocephalus ex vacuo	Anxiety
8	75	M	No *PTCH1* mutation	None	>15 active BCCs	Superficial bladder cancer
9	62	M	ND	None	>20 active BCCs Palmar and plantar pits	Prior NHL, psoriasis
10	72	M	ND	None	>5 active BCCs	Corneal ulcer, ectropion of eyelids

Abbreviations: UPN, unique patient number; FH, family history; AF, atrial fibrillation; DVT, deep venous thrombosis; HTN, hypertension; NHL, non-Hodgkin’s lymphoma. * internal deletion mutation ^†^ Family member genetically tested and determined to have PTCH1 mutation.

**Table 2:** Treatment characteristics.

UPN	Agent	Response (6 Months)	Toxicity	Schedule Modification	Treatment Duration (mo)	Escape	TTP (mo)	Outcome
1	S	CR	Muscle cramps, asthenia, taste changes	Yes, qod	5.4	none	5.4	New lesion resected
2	S	CR	Alopecia, weight loss	None *	95.8 *	localized	47.3	New lesion resected
3	S	CR	Taste changes, muscle cramps, diarrhea, hair thinning, nausea	Yes, 2 d/week	31.7	none	31.7	NED
4	S	CR	None	Yes, qod	5.7	none	5.7	NED
5	S	CR	Hair loss, muscle cramps, nausea	Yes, 5 d/week	28.4	localized	12.4	NED
6	S	CR	Muscle cramps, hair loss, nausea, vomiting	Yes *, qod	29.7 *	none	29.7	NED
7	V	CR	Hair thinning, taste changes, amenorrhea	None	80.9	localized	32.1	New lesion resected
8	V	CR	Muscle cramps, dysgeusia, weight loss, insomnia, stomach upset	Yes, 2 x/week	33.2	none	33.2	NED
9	V	CR	Muscle cramps, hair loss	None **	74.2 **	generalized	72.2	Died of AML
10	V	CR	Muscle cramps and taste changes	None	7.8	none	7.8	NED

Abbreviations: S, sonidegib; V, vismodegib; CR, complete response; NED, no evidence of disease; AML, acute myelogenous leukemia; TTP, time to progression. * Temporarily stopped taking medication due to co-payment cost/insurance. ** Temporarily stopped taking medication due to muscle cramps.

## Data Availability

The de-identified data underlying this case series will be shared upon reasonable request to corresponding authors.
